# Olfactory and Taste Disorders in Patients Suffering from Covid-19, a Review of Literature

**DOI:** 10.30476/DENTJODS.2021.87800.1284

**Published:** 2022-03

**Authors:** Kimia Ghods, Arezoo Alaee

**Affiliations:** 1 Student of Dentistry, Membership of Dental Material Research Center, Tehran Medical Sciences, Islamic Azad University, Tehran, Iran; 2 Dept. of Oral Medicine, Membership of Dental Material Research Center, Tehran Medical Sciences, Islamic Azad University, Tehran, Iran

**Keywords:** Anosmia, Ageusia, Dysgeusia, Covid-19, Coronavirus

## Abstract

Corona virus epidemic has caused a widespread disaster around the world. In studies, there are pieces of evidence of olfactory and taste dysfunction in patients with Covid-19. These symptoms occur independently or can be associated with other symptoms such as dry cough. The mechanism of the above-mentioned disorders and their clinical features in patients are not yet known. The rate of incidence of olfactory dysfunction in patients has been varied from 29.64% to 75.23% and the rate of incidence of taste dysfunction among the people can be different from 20.46% to 68.95%. Therefore, clinicians including ENT specialists and dentists should pay attention to the symptoms of anosmia and ageusia in these patients to prevent delayed diagnosis and inappropriate treatments. In this review article, data have been collected by searching the available articles in the domestic and foreign journals using databases such as PubMed, PubMed Central, Medline, EBSCO, Google Scholar, and Embase with key words of Anosmia, Ageusia, Dysgeusia, Covid-19, and Coronavirus from 2019 to 2020. Among the relevant references, 38 authoritative articles were chosen. The data showed that it seems olfactory and taste function disorders are the obvious symptoms of Covid-19, which can occur independently or with other symptoms, but the pathogenesis is not well specified yet. Therefore, further studies are required to achieve a reliable result in this area.

## Introduction

In December 2019, the corona virus broke out in Wuhan of China and spread rapidly throughout China and then all around the world. Therefore, World Health Organization called this disease as Covid-19 on February 12, 2020 [ 1 ].

In patients with Covid-19, the main symptoms include dry cough, shortage of breath and lymphocytopenia, along with the observation of a radiopaque examination on their chest CT-scan [ 2 ]. Patients with severe infections can also develop neurological symptoms such as skeletal muscle damage and memory impairment [ 3 ]. Among these symptoms, olfactory dysfunction (OD), including anosmia, hyposmia, as well as taste dysfunction, such as ageusia and dysgeusia, are especially prominent in patients with Covid-19 appear. The clinical examination of these symptoms has attracted much attention, because their evaluation does not require interventions, it can only be achieved with the help of questionnaires or screening algorithms. It is not clear whether olfactory and taste disorders are recurrent symptoms or are in the group of prognostic factors of this disease [ 4 ].

Many studies have been done around the world to examine these disorders. For example, recent studies in Iran showed a significant increase in the number of patients who have completely lost their sense of smell with the onset of Covid-19. Similar results can be seen in studies conducted in other countries of the world [ 5 ]. This article provides a review of the published articles related to these disorders. 

## Search Strategy

In this review, the databases such as PubMed, PubMed Central, Medline, EBSCO, Google Scholar, and Embase were used and 60 articles including 49 English articles and 9 Persian articles published between 2019 and 2020, which had one of the keywords such as Anosmia, Ageusia, Dysgeusia, Covid-19, Coronavirus in their titles were selected and studied. The inclusion criteria were the year of publication, the degree of relevance of the title and the purpose of the related articles. The exclusion criterion was excessive and non-relevant information. The researchers for the final analysis and writing of this review selected 33 English and 5 Persian articles. 

## Discussion

The corona virus family includes seven viruses, of which the SARS-Cov-2 virus is the pathogen for Covid-19. The receptor of this virus in the human body is called HACE2, and these receptors are located in the lungs, digestive system, nasal mucosa, urinary tract, lymphatic tissues, reproductive organs, vascular endothelium, brain, and kidneys. The virus is mainly enters the body through the nasal mucosa or digestive system due to the higher protein of HACE2 [ 6 ].

The symptoms of the disease appear when the virus binds to the receptor. The critical clinical manifestation of this disease is severe pneumonia, which is commonly reported in the elderly. The virus causes chronic inflammation of the lungs, severe shortage of breath, fever, dry cough, and cyanosis, and in vulnerable patients causes complete lung failure. Recent studies, however, have shown that patients with mild symptoms such as anosmia and other neurological problems indicate the aggressive neurological nature of the virus [ 7 ].

### The Relationship between Olfactory and Taste Disorders with Covid-19

Various documents indicate that olfactory disorder is one of the most prevalent symptoms of Covid-19 [ 8 ]. The olfactory and taste disorders have attracted the attention of ENT specialists around the world. A summary of published studies shows that the anosmia and ageusia are the evident symptoms of SARS-Cov-2 infection [ 9 ].

### Anosmia

It is described as the absence of the sense of smell, and the upper respiratory tract infection is its most common etiology. In addition, among the various pathogens, the most prevalent causes are viruses such as corona virus 229E and SARS-Cov [ 10 ]. However, the probability of occurrence of anosmia caused by SARS-Cov-2 may be much higher than SARS-Cov [ 1 ]. Patients suffering from Covid-19 may have a sudden onset of symptoms without any other symptoms, or may have other mild symptoms, such as dry cough, before it starts [ 11 - 12 ].

### Hyposmia

This means a decrease a sense of smell. Data from some various studies suggest that Covid-19 can lead to hyposmia in about 30% of patients. It is also observed in most patients that the olfactory disorder is not associated with nasal obstruction, so the probable sort of this disorder can be considered neurological [ 13 ].

### Ageusia

It is defined as a lack of sense of taste, which is seen in patients with Covid-19 and can appear alone or with other symptoms of the disease [ 14 ].

### Dysgeusia

It means taste failure, which is defined when a person has an unpleasant perception of a flavored substance and generally feels a metallic taste in his mouth [ 15 ].

The taste and smell disorders are observed in patients with Covid-19, and the presence or absence of these symptoms should be included in the evaluation and screening questionnaires of suspicious patients [ 16 ].

### Hypogeusia

This means a reduced ability to taste things. Some studies have stated that hypogeusia can be an initial presenting symptom of Covid-19 [ 17 ].

In 2020, a study was conducted by Lee *et al*. [ 14 ] on 3191 patients. This research showed that in 52% of the patients, ageusia and anosmia appeared simultaneously. Moreover, in 27.7% of patients only anosmia, and in 20.3% of patients only ageusia has been reported.They also reported that the average recovery time from anosmia in patients was 7 days, and most patients with anosmia or ageusia recovered within 3 weeks. In addition, young people, especially those in the age group of 20-39 years, showed a longer duration of anosmia [ 14 ].

Several cross-sectional studies have been conducted on the incidence of olfactory disorder in patients with Covid-19 in countries such as Italy [ 18 ], Iran [ 19 ], England [ 20 ], USA [ 21 ], Spain [ 22 ], France [ 23 ] and Germany [ 24 ], which showed that the incidence of olfactory disorder of patients with Covid-19 varies in different parts of the world (Table 1) [ 18 - 25 ]. Studies have also shown that patients with olfactory disorder tend to have a taste disorder, and there is a possible link between these two. In addition, the incidence of olfactory disorders is higher in women with Covid-19 than in men [ 25 ]. Therefore, the presence of anosmia or ageusia may be an important symptom of the differential diagnosis of Covid-19 [ 4 ]. 

**Table 1:** The incidence of Covid-19 patients with anosmia or dysgeusia in different countries

Country	Incidence
Spain [ 22 ]	83.9%
USA [ 21 ]	64.4%
UK [ 20 ]	59%
France [ 23 ]	45.3%
Italy [ 18 ]	33.9%
Germany [ 24 ]	13.5%
Iran [ 19 ]	10.6%

### Possible Hypotheses of Pathogenesis of Anosmia

The first possible hypothesis could be that the anosmia occurrence is due to damage caused by the virus to the olfactory pathways [ 26 ]. Evaluations performed on mouse models of SARS with anosmia showed a very low destruction of the olfactory epithelium [ 27 ]. Decreased lifespan of neurons in the epithelium is the most likely cause of decreased food support of the olfactory bulb of sensory neurons, and it can lead to mitral cell loss or lack of surviving mitral cell dendrites. Moreover, the virus may inflict its primary damage on the surface of the olfactory bulb rather than the epithelium, causing symptoms such as anosmia [ 27 ]. However, the corona viruses studied in these studies were specially modified in the laboratory, and this is not necessarily the case for SARS-Cov-2 [ 26 ].

However, recent reports of cases of Covid-19 indicate a high rate of recovery after the onset of olfactory dysfunction. Moreover, it seems that the frequency of central nervous system symptoms is about 25% lower than olfactory disorders in these patients [ 28 ]. Nerve cells may not be the main target of the virus, but non-neurons that include ACE2 receptors, such as the olfactory epithelium and olfactory bulbs, may be the main target of the virus [ 29 ].

The second possible hypothesis is based on a study conducted by Brann *et al*. [ 29 ]. This research concluded that infection of the epithelium and olfactory bulb cells caused by the virus could alter the function of the olfactory neurons and cause loss of the sense of smell. Stem cells involvement, which expresses lower levels of ACE2 receptors, can be the basis of long-term olfactory disorders [ 29 - 30 ].

### Possible Hypothesis of Pathogenesis of Ageusia

The first possible hypothesis of ageusia is since the ACE2 receptor is known as a cellular receptor for SARS-Cov-2 and is expressed diffusely on the mucous membrane of the oral cavity, especially on the tongue, and its role in modulating taste perception. It is now believed this receptor is involved in the development of taste disorders in Covid-19 [ 26 ].

The second possible hypothesis of ageusia is that the Middle East respiratory syndrome (MERS) corona virus may bind to sialic acid receptors [ 31 ]. This ability has also lately been seen in the pathogenesis process of SARS-CoV-2. Sialic acid is one of the elemental components of the salivary mucin, and protects glycoproteins, which carry gustatory molecules inside the taste buds from premature enzymatic degradation. Hence, SARS-CoV-2 could accelerate the degradation of the gustatory particles by occupying the binding sites of sialic acid on the taste buds [ 26 ].

### Diagnosis Tests Used for Olfactory and Taste Disorders

The first diagnosis test used for diagnosing Covid-19 patients with anosmia is olfactory function test. Olfactory function test has been introduced as the chief basis for the diagnosis of olfactory disorder disease [ 32 - 33 ].

In this test, which is performed in a quiet room, the patients’ olfactory threshold and their ability to distinguish various odors are evaluated. This test is performed by using an ortho-nasal olfactory device, which is a simple and reliable device. The evaluation of olfactory threshold is done with the help of butanol, by preparing eight bottles with different concentrations of n-butanol in deionized water. The maximum concentration of butanol is 4% placed in 60 milliliters of deionized water (bottle 0). Other bottles (from 1 to 8) have a diluted concentration of butanol. To perform the test, patients are provided with two identical impressible bottles, one containing n-butanol solution, and the other containing deionized water. The patients are then asked to close one nostril and immediately squeeze the bottle under their nostril and repeat this procedure for the next bottle. The patients are now asked to respond bottle smells more. If patients answer all four questions correctly, their olfactory function is presumed to be healthy. In case of error, the next bottle with the highest concentration of butanol is given to the patients and they are asked again. Finally, each of the two nostrils is assigned a score between 0 and 8 for the last bottle with the lowest concentration that has been correctly identified, and the average of these two is considered as the total score. To test the patient’s ability to distinguish different odors, common odors are placed in 180 milliliter opaque pots covered with gas and will be exposed to the patient at once in the same way as it was presented in the olfactory threshold assessment test. The patient is asked to identify the available fragrances, which include 10 items, thus score between 0 and 10 for each of the person’s nostrils, based on the number of correct odors guest, will be obtained. The 10 most common scents used in this test include baby powder, chocolate, coffee, ammonia, Vicks-VapoRub, fruit gum, ketchup, black pepper, soap, and orange [ 33 ].

The second diagnosis test used for diagnosing Covid-19 patients with dysgeusia is taste function test [ 33 ]. This test includes a standard and valid test that assesses the ability to perceive the four main flavors (sweet, salty, sour, and bitter), which is used to evaluate the taste function of patients with Covid-19. Initially, four solutions of each are prepared to measure each of the main flavors as follows: salt solution (30 grams of ordinary salt in one liter of deionized water), sweet solution (30 grams of refined sugar in one-liter deionized water), sour solution (90 milliliters of 100% commercial lemon juice in one liter of deionized water) and bitter solution (decaffeinated coffee and without sugar). It should be noted that deionized water is used as the control.

During the test, one milliliter of each solution is placed onto the patient’s tongue center. In addition, for each solution, a different swab is used. The patients are then asked to cite whether the perceived flavor is sweet, salty, bitter, sour, or neutral. In this experiment, solutions are given to the patient at random, and the only exception is the bitter solution, which is always presented last because it changes the patient’s perception of the next taste [ 33 ].

Finally, the patients’ answers are recorded as correct or incorrect. The score of each question varies from 0 to 4 and allows the patients to be classified into 4 groups as normal (score 4), mild hypogeusia (score 3), moderate hypogeusia (score 2), severe hypogeusia (score 1), and ageusia (score 0) [ 33 ].

### Treatments for Olfactory and Taste Disorders Associated with Covid-19

If olfactory and taste disorders persist for more than two weeks, it may be acceptable to consider a special treatment. The effectiveness of existing treatments for Covid -19 is unknown regarding olfactory disorders, but targeted disinfection therapies may be helpful for this disorder [ 34 ]. Frequent and intentional inhalation of a set of scents (lemon, rose, clove, and eucalyptus) is usually recommended twice a day, each time for at least 20 seconds for 3 months (if possible) since this method is effective, lost cost, and has few side effects [ 35 ].

Some medications including oral and intra-oral corticosteroids, alpha lipoic acid (600 mg/day), vitamin A (10000 U/day) and systemic omega 3 (2000 mg/day) can also be used to treat this disorder [ 36 - 37 ].

Despite all these cases, there is still no evidence that these treatments are effective in patients with olfactory disorder associated with Covid-19 [ 33 ]. There is a hypothesis for taste disorder, which states that changes in zinc cellular homeostasis in mouth taste cells are caused by the body’s immune response to SARS-Cov-2 viral replication, and thus by the creation of a hypozincemia mode, the person can suffer from dysgeusia. Therefore, according to this hypothesis, taking supplementary zinc pills or syrups (75 mg/day) can be effective as soon as dysgeusia symptoms appear [ 37 - 38 ].

### The Relationship between Olfactory and Taste Disorders with Dentistry

In 2020, research was done by Huaqiu Guo *et al*. [ 39 ] on 2537 dental patients. This study indicated that at the beginning of the Covid-19 outbreak, 38% patients went to the dental offices. The results of this study greatly suggest that Covid-19 highly influenced the smell and gustatory perception of the patients who visited these dental offices. Therefore, there are very strong documents, which show that in the post Covid-19 era, people with olfactory and gustatory disorders visit dental offices first. As a result, all the members of dental team should be trained and aware about these symptoms [ 40 ].

## Conclusion

Olfactory and taste disorders can be important symptoms of Covid-19, which can be seen solely or with other symptoms. The pathogenesis of this disease is not well specified and requires further future studies. Hence, the clinicians, dentists, and ENT specialists should pay attention to this important diagnostic symptom of the disease to prevent delayed diagnosis and subsequent possible inappropriate treatments in these patients. 

## Conflict of Interest

The authors declare that they have no conflict of interest.

## References

[1] Meng X, Deng Y, Dai Z, Meng Z ( 2020). COVID-19 and anosmia: A review based on up-to-date knowledge. Am J Otolaryngol.

[2] Wu Y, Xu X, Chen Z, Duan J, Hashimoto K, Yang L, et al ( 2020). Nervous system involvement after infection with COVID-19 and other coronaviruses. Brain Behav Immun.

[3] Koralnik I, Tyler K ( 2020). COVID ‐19: A Global Threat to the Nervous System. Ann Neurol.

[4] Gilani S, Roditi R, Naraghi M ( 2020). COVID-19 and anosmia in Tehran, Iran. Med Hypotheses.

[5] Carrillo-Larco R, Altez-Fernandez C ( 2020). Anosmia and dysgeusia in COVID-19: A systematic review. Wellcome Open Res.

[6] Hopkins C, Surda P, Whitehead E, Kumar B ( 2020 4). Early recovery following new onset anosmia during the COVID-19 pandemic- an observational cohort study. J Otolaryngol Head Neck Surg.

[7] Das G, Mukherjee N, Ghosh S ( 2020). Neurological Insights of COVID-19 Pandemic. ACS Chem Neurosci.

[8] Passali G, Bentivoglio A ( 2020). Comment to the article “Olfactory and gustatory dysfunctions as a clinical presentation of mild-to-moderate forms of the coronavirus disease (COVID-19): a multicenter European study”. Euro Arch Oto-Rhino-Laryngol.

[9] Heidari F, Karimi E, Firouzifar M, Khamushian P, Ansari R, Mohammadi Ardehali M, et al ( 2020). Anosmia as a Prominent Symptom of COVID-19 Infection. Rhinol J.

[10] Soler Z, Patel Z, Turner J, Holbrook E ( 2020). A primer on viral associated olfactory loss in the era of COVID‐19. Int Forum Allergy Rhinol.

[11] Gane S, Kelly C, Hopkins C ( 2020). Isolated Sudden Onset Anosmia in COVID-19 Infection. A Novel Syndrome?. Rhinol J.

[12] Eliezer M, Hautefort C, Hamel A, Verillaud B, Herman P, Houdart E, et al ( 2020). Sudden and Complete Olfactory Loss of Function as a Possible Symptom of COVID-19. JAMA Otolaryngol Head Neck Surg.

[13] Bocksberger S, Wagner W, Hummel T, Guggemos W, Seilmaier M, Hoelscher M, et al ( 2020). Temporäre Hyposmie bei COVID-19-Patienten. HNO.

[14] Lee Y, Min P, Lee S, Kim SW ( 2020). Prevalence and Duration of Acute Loss of Smell or Taste in COVID-19 Patients. J Korean Med Sci.

[15] Mahmoud MM, Abuohashish HM, Khairy DA, Bugshan AS, Khan AM, Moothedath MM ( 2021). Pathogenesis of dysgeusia in COVID-19 patients: a scoping review. Eur Rev Med Pharmacol Sci.

[16] Aziz M, Perisetti A, Lee-Smith WM, Gajendran M, Bansal P, Goyal H ( 2020). Taste Changes (Dysgeusia) in COVID-19: A Systematic Review and Meta-analysis. Gastroenterology.

[17] Melley LE, Bress E, Polan E ( 2020). Hypogeusia as the initial presenting symptom of COVID-19. BMJ Case Rep.

[18] Giacomelli A, Pezzati L, Conti F, Bernacchia D, Siano M, Oreni L, et al ( 2020). Self-reported Olfactory and taste disorders in patients with severe acute respiratory coronavirus 2 infection: a cross-sectional study. Clin Infect Dis.

[19] Bagheri S H, Asghari A, Farhadi M, Shamshiri A R, Kabir A, Kamrava S K, et al ( 2020). Coincidence of COVID-19 epidemic and olfactory dysfunction outbreak in Iran. Med J Islam Repub Iran.

[20] Menni C, Valdes A, Freydin MB, Ganesh S, El-Sayed Moustafa J, Visconti A, et al Loss of smell and taste in combination with other symptoms is a strong predictor of COVID-19 infection. https://www.medrxiv.org/content/10.1101/2020.04.05.20048421v1.

[21] Yan C, Faraji F, Prajapati D, Boone C, DeConde A ( 2020). Association of chemosensory dysfunction and COVID‐19 in patients presenting with influenza‐like symptoms. Int Forum Allergy Rhinol.

[22] Barón-Sánchez J, Santiago C, Goizueta-San Martín G, Arca R, Fernández R ( 2020). Smell and taste disorders in Spanish patients with mild COVID-19. Neurologia.

[23] Zayet S, Kadiane-Oussou N, Lepiller Q, Zahra H, Royer P, Toko L, et al ( 2020). Clinical features of COVID-19 and influenza: a comparative study on Nord Franche-Comte cluster. Microb Infect.

[24] Seidel M, Hölzer B, Appel H, Babel N, Westhoff T ( 2020). Impact of renal disease and comorbidities on mortality in hemodialysis patients with COVID-19: a multicenter experience from Germany. J Nephr.

[25] Spinato G, Fabbris C, Polesel J, Cazzador D, Borsetto D, Hopkins C, et al ( 2020). Alterations in smell or taste in mildly symptomatic outpatients with SARS-CoV-2 infection. JAMA.

[26] Vaira L, Salzano G, Fois A, Piombino P, De Riu G ( 2020). Potential pathogenesis of ageusia and anosmia in COVID‐19 patients. Int Forum Allergy Rhinol.

[27] Butowt R, von Bartheld C ( 2020). Anosmia in COVID-19: underlying mechanisms and assessment of an olfactory route to brain infection. Neuroscientist.

[28] Mao L, Jin H, Wang M, Hu Y, Chen S, He Q, et al ( 2020). Neurologic manifestations of hospitalized patients with coronavirus disease 2019 in Wuhan, China. JAMA Neurol.

[29] Brann D, Tsukahara T, Weinreb C, Lipovsek M, Van den Berge K, Gong B, et al ( 2020). Non-neuronal expression of SARS-CoV-2 entry genes in the olfactory system suggests mechanisms underlying COVID-19-associated anosmia. Sci Adv.

[30] Moein S, Hashemian S, Mansourafshar B, Khorram‐Tousi A, Tabarsi P, Doty R ( 2020). Smell dysfunction: a biomarker for COVID‐19. Int Forum Allergy Rhinol.

[31] Park YJ, Walls AC, Wang Z, Sauer MM, Li W, Tortorici MA, et al ( 2019). Structures of MERS-CoV spike glycoprotein in complex with sialoside attachment receptors. Nat Struct Mol Biol.

[32] Ottaviano G, Carecchio M, Scarpa B, Marchese-Ragona R ( 2020). Olfactory and rhinological evaluations in SARS-CoV-2 patients complaining of olfactory loss. Rhino J.

[33] Vaira L, Deiana G, Fois A, Pirina P, Madeddu G, De Vito A, et al ( 2020). Objective evaluation of anosmia and ageusia in COVID ‐19 patients: Single‐center experience on 72 cases. Head Neck.

[34] Whitcroft KL, Hummel T ( 2020). Olfactory Dysfunction in COVID-19: Diagnosis and Management. JAMA.

[35] Whitcroft K, Hummel T ( 2019). Clinical Diagnosis and Current Management Strategies for Olfactory Dysfunction. JAMA Otolaryngol Head Neck Surg.

[36] Yan CH, Rathor A, Krook K, Ma Y, Rotella MR, Dodd RL, et al ( 2020). Effect of Omega-3 Supplementation in Patients With Smell Dysfunction Following Endoscopic Sellar and Parasellar Tumor Resection: A Multicenter Prospective Randomized Controlled Trial. Neurosurg.

[37] Miwa T, Ikeda K, Ishibashi T, Kobayashi M, Kondo K, Matsuwaki Y, et al ( 2019). Clinical practice guidelines for the management of olfactory dysfunction- Secondary publication. Auris Nasus Larynx.

[38] Lozada-Nur F, Chainani-Wu N, Fortuna G, Sroussi H ( 2020). Dysgeusia in COVID-19: possible mechanisms and implications. Oral Surg Oral Med Oral Pathol Oral Radiol.

[39] Guo H, Zhou Y, Liu X, Tan J ( 2020). The impact of the COVID-19 epidemic on the utilization of emergency dental services. J Dent Sci.

[40] Baghizadeh Fini M ( 2020). What dentists need to know about COVID-19. Oral Oncol.

